# Dietary Conjugated Linoleic Acid (CLA) increases milk yield without losing body weight in lactating sows

**DOI:** 10.1186/2055-0391-56-11

**Published:** 2014-07-30

**Authors:** Sung-Hoon Lee, Young-Kuk Joo, Jin-Woo Lee, Young-Joo Ha, Joon-Mo Yeo, Wan-Young Kim

**Affiliations:** Livestock Experiment Station, Gyeongsangnamdo Livestock Promotion Research Institute, 251 Cheonghyun-ro, Sinan-myeon, Sancheong, 666-962 Republic of Korea; Department of Beef & Dairy Science, Korea National College of Agriculture and Fisheries, 212 Hyohaeng-ro, Bongdam-eup, Hwaseong, 445-760 Republic of Korea

**Keywords:** Conjugated linoleic acid, Milk yield, Body weight, Sows, Piglets

## Abstract

This study was conducted to evaluate the effects of dietary conjugated linoleic acid (CLA) on the performance of lactating sows and piglets as well as the immunity of piglets suckling from sows fed CLA. Eighteen multiparous Duroc sows with an average body weight (BW) of 232.0 ± 6.38 kg were randomly selected and assigned to two dietary treatments (n = 9 for each treatment), control (no CLA addition) and 1% CLA supplementation. For the control diet, CLA was replaced with soybean oil. Experimental diets were fed to sows during a 28-day lactation period. Litter size for each sow was standardized to nine piglets by cross-fostering within 24 hours after birth. Sow milk and blood samples were taken from sows and piglets after 21 and 27 days of lactation, respectively. Loss of BW was significantly (p < 0.05) higher in sows fed control diet compared to sows fed CLA diet. Piglet weights at weaning and weight gain during suckling were significantly (p < 0.05) higher in sows fed CLA compared to sows fed control diet. Serum non-esterified fatty acid (NEFA) and urea nitrogen concentrations were significantly (p < 0.05) lower in sows fed CLA than in sows fed soybean oil. IgG concentrations of the groups supplemented with CLA increased by 49% in sow serum (p < 0.0001), 23% in milk (p < 0.05), and 35% in piglet serum (p < 0.05) compared with the control group. Sows fed CLA showed an increase of 10% in milk yield compared with sows fed soybean oil (p < 0.05), even though there was no difference in daily feed intake between the treatments. Milk fat content was significantly (p < 0.05) lower in sows fed CLA than in sows fed soybean oil. Solid-not-fat yield was significantly (p < 0.05) higher in sows supplemented with CLA than in sows fed control diet and also protein-to-fat ratio in milk was significantly (p < 0.05) higher in sows fed CLA compared with the control group. The results show that CLA supplementation to sows increased milk yield without losing BW during lactation, whereas soybean oil supplementation resulted in severe BW loss.

## Background

The growth rate of suckling piglets is determined by the amount of milk produced by sows [1]. Greater milk production in sows increases pig weaning weights as well as viability of offspring, and pigs with heavier weaning weights grow more rapidly at post-weaning [2, 3]. It has been well documented that sows mobilize sufficient energy from their body tissue stores for milk production [4–6]. Deprivation of milk from sows has been shown to reduce body weight (BW) of sows during lactation [7]. Further, several studies have reported that a BW loss between 10 to 12% during lactation reduced reproductive performance in the subsequent parity [8, 9]. Thus, it is important to minimize BW loss in sows during lactation as well as maintain both maximal growth of piglets and subsequent reproductive performance.

To solve this problem, it was intended to feed lactating sows a diet containing conjugated linoleic acid (CLA), which is composed of an isomeric mixture of linoleic acid containing conjugated double bonds, predominantly *cis*-9, *trans*-11 CLA (c9, t11) and *trans*-10, *cis*-12 CLA (t10, c12), produced from polyunsaturated fatty acids by ruminal bacteria during biohydrogenation [10]. CLA has been extensively studied due to its beneficial effects on humans and animals [11, 12]. Consumption of CLA by sows during lactation has been found to lower backfat thickness loss as well as increase weaning weight in piglets [13, 14]. Higher BW at weaning is closely associated with higher milk yield [6, 15]. In contrast, Harrell et al. [16] and Peng et al. [17] showed that inclusion of CLA did not affect piglet weights at weaning. As these results are contradictory, further research is needed to elucidate the effects of dietary CLA on milk yield and body weight changes in lactating sows. Regarding immunity in piglets, Corino et al. [13, 18] found that increasing levels of CLA fed to sows or weaned piglets markedly increased the immunoglobulin G (IgG) concentrations of piglets during suckling and post-weaning. Therefore, transfer of CLA from sows to milk could reduce mortality in piglets by increasing immunity.

The objective of this study was to determine the effects of dietary CLA on the performance of lactating sows and piglets as well as blood and milk compositions. It further examined the effects of CLA on IgG concentrations in sera from sows and piglets, and milk.

## Methods

The animal use and care protocol was approved by the Institutional Animal Care and Use Committee of the Gyeongsangnamdo Livestock Promotion Research Institute, Korea.

### Animal and experimental diets

Eighteen multiparous Duroc sows in their 2nd to 5th parities with an average body weight (BW) of 232.0 ± 6.38 kg were randomly selected and assigned to two dietary treatments (n = 9 for each treatment), control (0% CLA) and 1% CLA addition. CLA was replaced with soybean oil in the control diet. The sows were moved into farrowing rooms after 108 days of gestation and were housed individually in crates (2.4 m × 1.7 m) with slatted floors.

Experimental diets were provided in the form of flour and were formulated to meet the recommended amounts of crude protein and digestible energy (DE) as required by the NRC [19]. The two diets were isoenergetic and isonitrogenous. Ingredients and chemical compositions of the experimental diets are shown in Table 1. Diets were provided from 110 days of gestation until weaning (28 days postpartum). Sows were fed twice daily at approximately 07:00 and 17:00 h and had *ad libitum* access to water. The diets were restricted to 2.0 kg/day for each animal and were administered 24–48 hours prior to farrowing in order to prevent excessive gut fill from obstructing the farrowing process *per se* as well as to minimize any problem with mastitis-metritis-agalactia. After farrowing, sows were initially fed 1.5 kg of their treatment diet twice daily (08:00 and 16:30 h), and this was increased daily by 0.5 kg until 7 days postpartum. Thereafter, sows had free access to their diets until weaning.

**Table 1:** **Ingredients and chemical compositions of experimental diets**

	Sow diets	
	CON ^1^	CLA ^2^
Ingredients, %		
Corn	45.60	45.60
Wheat	8.00	8.00
Wheat bran	5.00	5.00
Rice bran	12.25	12.25
Soybean meal	16.00	16.00
Rape seed meal	5.00	5.00
Palm oil meal	1.50	1.50
Cotton seed meal	1.00	1.00
Soybean oil^3^	1.00	0.00
Conjugated linoleic acid oil^4^	0.00	1.00
Monocalcium phosphate	0.10	0.10
Limestone	1.67	1.67
Tallow	2.00	2.00
Sodium chloride	0.60	0.60
Vitamin and mineral premix^5^	0.20	0.20
L-lysine HCl	0.08	0.08
Total	100.00	100.00
Chemical composition		
Moisture, %	12.28	12.27
Crude protein, %	17.48	17.10
Crude fat, %	9.38	9.84
Crude fiber, %	4.58	4.60
Crude ash, %	5.53	5.56
Calcium, %	50.75	50.63
Phosphorus, %	0.93	0.90
Lysine, %	0.53	0.51
Nitrogen-free extracts, %	0.86	0.85
Digestible energy, kcal/kg^6^	3,393	3,393

^1^CON = diets supplemented with soybean oil.

^2^CLA = diets replaced soybean oil with CLA.

^3^Soybean oil, purchased from Cheiljedang (Seoul, Korea), contains C16:0 10.92%; C18:0 4.20%; C18:1*cis*-9 24.04%; C18:2*n*-6 54.38%; and C18:3*n*-36.46%.

^4^Conjugated linoleic acid oil, purchased from HK Biotech (Jinju, Korea), contains C16:0 6.57%; C18:0 2.45%; C18:1*cis*-9 10.01%; C18:2*n*-6 1.76%; CLA *cis*-9, *trans*-11 33.91%; and CLA *trans*-10, *cis*-12 41.47%.

^5^Vitamin-mineralpremix provides per kg of feed: vitamin A 10,000IU; vitamin D_3_ 2,000IU; vitamin E 44 mg; vitamin K_3_ 2 mg; vitamin B_1_ 1.3 mg; vitamin B_2_ 4.0 mg; vitamin B_6_ 1.3 mg; vitamin B_12_ 0.015 mg; pantothenic acid 12 mg; nicotinic acid 20 mg; biotin 0.2 mg; folic acid 1.3 mg; iron 80 mg; copper 5 mg; cobalt 0.30 mg; zinc 50 mg; manganese 20 mg; iodine 0.14 mg.

^6^Calculated value.

To evaluate sow performance, litter sizes were adjusted (nine piglets per sow) by cross-fostering piglets within 24 hours after birth. Piglets had no access to creep feed. At birth, the litters were subjected to normal management procedures, including cutting of teeth and tails, ear notching, and iron shots. Males were not castrated.

All weaned piglets were mixed across treatments and then moved to pens (10 piglets per pen, arranged based on similar BW) in an environmentally controlled room. Piglets were raised on the same commercial diet (PuKyung Pig Farmers Agricultural Cooperative Feed Mill, Gimhae, Korea) until 70 days of age. Piglets after weaning were separated into pre-starter and starter phases, respectively. Pre-starter diet consisting of 19% crude protein, 1.3% lysine, 3,500 kcal/kg DE, 0.76% calcium, and 0.62% phosphorus was given from 29–49 days, followed by starter diet consisting of 17.5% crude protein, 1.0% lysine, 3,350 kcal/kg DE, 0.67% calcium, and 0.57% phosphorus until 70 days of age.

### Measurements and sampling

The BWs of sows were recorded at farrowing and weaning (28 days), and differences in BW were calculated. For piglets, birth and weaning weights were also recorded, and daily BW gain was calculated. After weaning, individual BWs of piglets were recorded at 70 days of age. Average daily gain was calculated during the 42-day post-weaning period and total period (0 to 70 days of age). Backfat thickness of sows was measured by ultrasound at farrowing and weaning using a Renco Lean Meater® (Renco Corporation, Minneapolis, USA). Measurements were taken 65 mm from the midline at the last rib. Feed intake during lactation was recorded daily. The number of pigs that died during the lactation period was counted, and mortality (%) was calculated. Weaning-to-estrus interval was determined by monitoring estrus from 3 to 10 days after weaning.

On day 21 of lactation, milk samples were collected. Litters were separated from sows for 1 hour prior to milking, after which approximately 50 mL of milk was obtained after intramuscular injection of 20 IU of oxytocin (Komioxytocin inj.; Komipharm International Co., Ltd., Siheung, Korea). Milk samples were then frozen immediately at -80°C for milk composition and IgG analysis. Blood samples taken from the jugular vein were collected from sows prior to feeding in the morning on the day before weaning. Three randomly selected piglets per sow were then subjected to blood collection from the anterior vena cava, after which blood samples were pooled within the same litter. Before collecting blood samples, suckling piglets were not segregated from sows. Serum was separated by centrifugation (3,000 × g for 15 min at 4°C) and frozen immediately at -80°C until analyses.

Milk production by sows was measured on day 21 of lactation by a modified weigh-suckle-weigh (WSW) method of Speer and Cox [15]. Briefly, litters were separated from their dam for 1 hour. Piglets were placed in a pen under a heat lamp during separation. Litters then were weighed to obtain their pre-suckling BW, returned to their mothers, allowed to suckle until the end of vigorous synchronized suckling by the litter, and then immediately collected and weighed to obtain their post-suckling BW. This procedure was repeated hourly until a minimum of three consistent measurements of hourly milk yield were obtained. Hourly milk yields were a measurement of BW gain, as litter milk intake was based on the difference between pre- and post-suckling litter BWs. Mean hourly milk yield multiplied by 24 was used to estimate daily milk yield. Suckling frequency was not controlled on the other days of lactation.

### Sample analyses

Compositional analysis of the experimental diets was carried out according to the procedures of AOAC [20]. Fatty acids of the two oil sources (soybean oil and CLA oil) used in this study were analyzed by the one-step procedure described by Sukhija and Palmquist [21]. Serum glucose content was determined using an enzymatic kit (Glucose Hexokinase kit, Bayer, US). Serum total protein and urea nitrogen contents were determined using an auto analyzer (model 704, Hitachi). Serum total cholesterol, triacylglyceride, and non-esterified fatty acid (NEFA) levels were determined by enzymatic spectrophotometric assay (Boehringer Mannheim, Germany). Serum total lipid content was determined by colorimetric assay (Hitachi 7180, Japan). Serum LDL- and HDL-cholesterol levels were determined by enzymatic colorimetric assay (Roche, Germany). IgG concentrations of serum and milk were determined by the radial immunodiffusion method of Mancini et al. [22] using a commercial kit (Bethyl Laboratories Inc., Montgomery, TX). Serum thyroxine (T4) and triiodothyronine (T3) levels were measured using radioimmunoassay (RIA) kits (ICN Pharmaceuticals, Inc., Costa Mesa, CA). Analyses for T4 and T3 levels were performed in duplicate. Milk composition (%) was analyzed using Milkoscan FT 120 (FOSS Electric, Korea).

### Statistical analysis

Data were analyzed by t-test for a completely randomized design using the GLM procedure of SAS [23]. Least squares means were calculated for each independent variable. Individual sows and their litters were used as the experimental unit. In weaned piglets, gender effect was ignored and thus not included in the model. Differences were considered significant at *p* < 0.05.

## Results

### Sow performance

Results on BW, backfat thickness, total feed intake, and weaning-to-estrus interval of sows during lactation are shown in Table 2. Loss of BW during the lactation period was significantly (*p* < 0.05) higher in sows fed control diet compared to sows fed CLA diet. Sows fed CLA diet showed little change in BW during lactation. No difference in backfat thickness, total feed intake, or weaning-to-estrus interval was observed between the treatments during the lactation period.

**Table 2:** **Body weight, backfat thickness, total feed intake, and weaning-to-estrus interval in lactating sows fed diets supplemented with CLA**

	Sow diets		SED ^3^	***p***-value
	CON ^1^	CLA ^2^		
No. of sows	9	9		
Parity	3.08	3.09	0.57	0.9896
Body weight, kg				
24 h postpartum	232.19	231.82	13.16	0.9781
Weaning (day 28)	222.16	231.52	11.83	0.4401
Difference	-10.03	-0.31	4.26	0.0364
Backfat thickness, mm				
24 h postpartum	15.56	15.33	1.37	0.8727
Weaning (day 28)	14.30	14.97	1.35	0.6295
Difference	-1.26	-0.36	0.85	0.3095
Total feed intake, kg	181.36	184.16	2.20	0.2179
Weaning-to-estrus interval, day	4.22	4.78	0.46	0.2536

^1^CON = diets supplemented with soybean oil.

^2^CLA = diets replaced soybean oil with CLA.

^3^SED = standard error of difference.

### Performances of litters and piglets

Performances of litters and piglets during the 28-day lactation period are presented in Table 3. Dietary CLA had no effect on the number of weaned piglets or piglet mortality during lactation. Litter weight at weaning (*p* = 0.0722) and daily litter weight gain (*p* = 0.096) tended to be higher in sows fed CLA diet compared with sows fed control diet. Furthermore, piglet weight at weaning was significantly (*p* < 0.05) increased by sow consumption of CLA diet, but this was not evident after 42 days of weaning. Piglet weight gain was significantly (*p* < 0.05) higher in sows fed CLA diet during the suckling period, whereas it was unaffected from weaning until 70 days of age as well as birth to 70 days of age.

**Table 3:** **Performances of litters and piglets in lactating sows fed diets supplemented with CLA**

	Sow diets		SED ^3^	***p***-value
	CON ^1^	CLA ^2^		
Litter size, no. of piglets				
After cross-fostering (day 0)	9	9		
At weaning (day 28)	8.71	8.63	0.26	0.7377
Pre-weaning mortality, %	3.17	4.17	2.90	0.7377
Litter weight, kg				
After cross-fostering (day 0)	14.45	14.87	1.11	0.7097
At weaning (day 28)	62.88	69.14	3.20	0.0722
Gain (day 0 to 28), kg/d	1.73	1.94	0.12	0.0960
Piglet weight, kg				
After cross-fostering (day 0)	1.60	1.65	0.12	0.6965
At weaning (day 28)	7.21	8.03	0.33	0.0278
42 days post-weaning (day 70)	24.54	25.98	1.03	0.1841
Piglet weight gain, g/day				
Day 0 to 28	199.94	227.75	11.21	0.0276
Day 28 to 70	412.46	431.51	23.07	0.4239
Day 0 to 70	327.67	347.57	14.25	0.1859

^1^CON = diets supplemented with soybean oil.

^2^CLA = diets replaced soybean oil with CLA.

^3^SED = standard error of difference.

### Blood metabolites, thyroid hormones, and IgG concentrations

Results on blood metabolites, thyroid hormones, and IgG concentrations in blood and milk are shown in Table 4. Blood metabolite levels of sows were unaffected, whereas NEFA and urea nitrogen concentrations were significantly (*p* < 0.05) reduced by CLA diet. There was no significant difference in T3 or T4 concentration between the treatments. IgG concentrations were significantly higher in sera of sows (*p* < 0.01) and piglets (*p* < 0.05) fed CLA diet as well as milk (*p* < 0.05) of sows fed CLA diet compared to sows fed control diet.

**Table 4:** **Serum concentrations of metabolites and thyroid hormone in lactating sows fed control diet or CLA diet, and serum and milk IgG concentrations of sows and their progeny**

	Sow diets		SED ^3^	***p***-value
	CON ^1^	CLA ^2^		
Sow serum characteristics				
Glucose, mg/dL	72.42	76.73	5.46	0.4586
Total protein, g/dL	7.19	7.11	0.17	0.6358
Total lipid, mg/dL	225.50	191.50	24.13	0.2085
Triacylglycerol, mg/dL	19.33	21.36	3.07	0.5151
NEFA, μEq/L	306.50	170.44	64.77	0.0484
Total cholesterol, mg/dL	97.50	99.91	6.58	0.7180
LDL-cholesterol, mg/dL	50.33	49.40	4.08	0.8213
HDL-cholesterol, mg/dL	49.75	47.46	4.47	0.6130
HDL/Total cholesterol	0.51	0.49	0.04	0.6007
LDL/HDL cholesterol	1.03	1.08	0.16	0.7708
Urea nitrogen, mg/dL	13.49	11.60	0.66	0.0134
Thyroxine (T4), μg/dL	2.88	3.01	0.18	0.4661
Triiodothyronine (T3), ng/mL	0.61	0.67	0.05	0.1729
T3:T4, %	2.15	2.24	0.16	0.5722
IgG, mg/dL				
Sow serum	596.42	889.33	41.54	<0.0001
Milk	42.16	52.06	4.11	0.0468
Piglet serum	187.78	253.88	26.48	0.0189

^1^CON = diets supplemented with soybean oil.

^2^CLA = diets replaced soybean oil with CLA.

^3^SED = standard error of difference.

### Milk yield and composition

Milk yield and composition in sows fed CLA diet during lactation are shown in Table 5. Sows fed CLA diet showed a significantly (*p* < 0.05) higher milk yield than those fed control diet, even though there was no significant difference in daily lactational feed intake between the treatments. Fat content was significantly (*p* < 0.05) lower in sows fed CLA diet than in those fed control diet. However, there were no differences in other milk components by dietary CLA consumption during lactation. Except for lactose and solid-not-fat yields, there were no differences in yields of other components upon dietary CLA consumption. Yield of lactose tended (*p* = 0.0532) to increase in sows fed CLA diet compared with sows fed control diet, and solid-not-fat yield was significantly (*p* < 0.05) higher in sows supplemented with CLA compared to those fed control diet. Further, protein-to-fat ratio was significantly (*p* < 0.05) higher in sows fed CLA diet compared with sows fed control diet.

**Table 5:** **Daily lactational feed intake, milk yield, and composition in lactating sows fed control diet or CLA diet**

	Sow diets		SED ^3^	***p***-value
	CON ^1^	CLA ^2^		
Lactational feed intake, kg/day	6.48	6.58	0.08	0.2117
Milk yield at 21 days of lactation, kg/day	8.54	9.49	0.39	0.0288
Fat				
%	8.44	5.92	1.05	0.0308
Yield, kg/day	0.72	0.56	0.10	0.1158
Protein				
%	5.15	4.80	0.28	0.1838
Yield, kg/day	0.44	0.46	0.02	0.5033
Lactose				
%	5.19	5.44	0.18	0.1855
Yield, kg/day	0.45	0.52	0.03	0.0532
Solids not fat				
%	10.93	10.92	0.24	0.9492
Yield, kg/day	0.93	1.04	0.05	0.0367
Protein: fat	0.64	0.85	0.09	0.0288

^1^CON = diets supplemented with soybean oil.

^2^CLA = diets replaced soybean oil with CLA.

^3^SED = standard error of difference.

## Discussion

In the present study, sows fed CLA showed little change in BW during the lactation period. This result is in disagreement with previous studies that have reported no dietary CLA effect on BW change in sows [13, 16, 24]. In contrast, sows fed control diet containing soybean oil (high in linoleic acid (LA); Table 1) at the expense of CLA showed severe loss of BW. It seems that lactating sows that consume a diet containing soybean oil rather than CLA oil more readily mobilize energy from their body reserves to produce milk. This may be related to the acceleration of body fat catabolism. Sanz et al. [25] reported that broiler chickens fed sunflower oil diet, which is also high in LA, had higher specific activities of fat-catabolic enzymes such as carnitine palmitoyltransferase I (CPT I) and L-3-hydroxyacyl-CoA dehydrogenase (L3HOAD). Likewise, Shimomura et al. [26] observed lower fat deposition in rats fed a diet rich in safflower oil compared to those fed tallow. However, Vicente et al. [27] found that fat sources had no effect on BW of lactating sows, which contradicts the results of the present study.

In our study, there was no significant difference in backfat thickness between the treatments. In support of our result, Harrell et al. [16] previously reported no difference in backfat thickness, whereas Cordero et al. [14] showed a less loss of backfat thickness in sows fed CLA diet. Conversely, Park et al. [28] reported that backfat thickness became thinner at a higher level of CLA supplementation or longer feeding time in lactating sows. Meanwhile, in grower-finisher pigs, many researchers reported that CLA treatment reduced backfat thickness [29–33]. These contradictory results on CLA feeding may be due to the different physiological state of swine. Therefore, the backfat thickness of sows fed CLA during lactation must be further investigated.

In the present study, BW changes during lactation did not affect the subsequent weaning-to-estrus interval. Thus, it seems that BW loss was not sufficiently low enough to reduce subsequent reproductive performance. On the other hand, Reese et al. [34] demonstrated that little relationship existed between sow weight loss during lactation and the interval between weaning and first estrus. Total feed intake of sows was not influenced by dietary inclusion of CLA, in accordance with previous studies [13, 14, 16, 24].

Litter size at weaning and mortality of suckling piglets were not affected by CLA supplementation. This result is in agreement with those of Cordero et al. [14], who observed no effect of dietary CLA on the number of weaned piglets or piglet mortality. Reduction of litter size at weaning and mortality during the suckling period may be dependent on numerous factors, such as milk quality of sows, suckling intensity, disease, accidents, environmental conditions, and unknown factors [35].

Although litter weight was maintained after cross-fostering piglets, weaning litter weight as well as litter weight gain increased in the CLA dietary group (Table 3), reflecting increased milk yield by sows [6, 36]. Moreover, weaning litter weight and weight gain were drastically elevated in piglets from sows fed CLA compared to those from sows fed soybean oil, even though piglet weights at birth were similar between the treatments. These results corroborate the published data of Corino et al. [13] and Cordero et al. [14], who reported that piglets from sows fed a diet supplemented with 0.5% or 1% CLA during lactation were markedly heavier than piglets from control sows. Cabrera et al. [37] further demonstrated a link between higher weaning weight and reduced time to reach finishing weight in pigs, which implies market pigs (BW = 110 kg) can be produced more economically. However, maternal CLA background had no effect on piglet weights or weight gain throughout the 42-day post-weaning period, which is in agreement with the observations of Bontempo et al. [24] and Corino et al. [13]. On the other hand, Bee [38] observed that, irrespective of starter diet, pigs reared by sows fed CLA during lactation showed greater feed intake, weight gain, and final weights after weaning than pigs reared by sows fed LA diet. These discrepancies might be due to the higher level of CLA (2%) used by Bee [38].

It was previously reported that CLA reduces body fat content [39]. In the present study, serum NEFA concentration was drastically reduced in sows fed CLA compared with sows fed soybean oil, suggesting the reduction of body fat mobilization. Further, a lower NEFA concentration is consistent with higher BW of sows, as shown in Table 2. In contrast, Bontempo et al. [24] and Corino et al. [13] reported that 0.5% CLA supplementation to sows during lactation had no effect on NEFA levels. Corino et al. [18] also observed no difference in NEFA concentration with increasing amount of CLA, whereas triacylglycerol levels markedly decreased in rabbits. On the other hand, Ostrowska et al. [40] found that dietary CLA treatment significantly increased plasma NEFA and triacylglycerol levels in pigs, with no effect on plasma glucose and insulin levels. In addition, Gutgesell et al. [41] revealed that lactating rats fed CLA diets showed a greater concentration of NEFA in plasma compared to rats fed diets containing sunflower oil.

As shown in this study, dietary CLA had no effect on total protein, total lipid, or triacylglycerol concentration, which is in accordance with the results of Stangl [42, 43]. The glucose concentration also did not differ between the treatments. This result corroborates data from Bontempo et al. [24] and Corino et al. [13], who reported that the serum glucose concentration of lactating sows remained unchanged after CLA treatment. Ramsay et al. [44] also found that CLA supplementation up to 2% had no effect on the serum glucose concentration of growing pigs. On the contrary, Stangl [42] found that rats fed 5% CLA exhibited a higher concentration of glucose than control rats.

In the present study, CLA had no effect on total, LDL-, or HDL-cholesterol concentration, which leads to no difference in the HDL-to-total cholesterol or LDL-to-HDL-cholesterol ratio. There is little information on the effects of dietary CLA on the serum cholesterol profile of lactating sows. Stangl [42] found that rats fed 5% CLA showed significantly reduced total, LDL-, and HDL-cholesterol levels, whereas rats fed less than 5% CLA showed no differences. Mele et al. [45] also reported that dietary intake of 0.8 g of *cis*-9, *trans*-11 CLA per day in humans tended to reduce the plasma LDL-cholesterol level. Stangl [43] also reported that the serum cholesterol level of growing rats fed 3% CLA under conditions of enhanced fat mobilization remained unchanged. Moreover, Stangl et al. [46] observed no difference in the serum concentration of total, LDL-, or HDL-cholesterol in adult female pigs fed CLA at a dietary level of 1%, which is in accordance with data from this study. On the other hand, Corino et al. [18] found that total cholesterol level in rabbits was markedly reduced by consumption of 0.25 or 0.5% CLA. Nunes et al. [47] also reported that dogs fed 0.5% CLA for nine months showed a 34% reduction in the total cholesterol level, as well as 28% reduction in the levels of LDL and non-HDL-cholesterol. Therefore, CLA can have differential effects depending on its level, dominant isomer, animal species used, and physiological status.

The present data show that the serum urea nitrogen concentration was remarkably higher in sows fed soybean oil compared to those fed CLA. This result may be due to higher catabolism of body protein during lactation in sows fed soybean oil compared to those fed CLA.

The present study examined the thyroid hormones T4 and T3 as an indicator of mammary gland growth and development [48]. In the present study, there was no difference in the T4 or T3 concentration between the treatments. This result is in agreement with data from Stangl et al. [46] and Bontempo et al. [24]. In contrast, Corino et al. [13] found that sows fed CLA until weaning had a high concentration of serum thyroxine, resulting in heavier piglets via increased milk yield. Beckett et al. [49] found that conversion of T4 to T3, the biologically active form of thyroid hormone, is controlled by selenium-containing enzymes. Consequently, the present study again confirmed no relationship between CLA and T3.

As in other species, IgG is the most representative antibody in pig serum, and its concentration is an index of antibody production against antigenic stimuli not processed by T-cells. In the present study, the IgG concentrations of sows fed CLA increased by 49% in sow serum, 23% in milk, and 35% in piglet serum compared with levels of control groups. Furthermore, piglets from sows fed CLA diet also showed increased serum IgG concentrations. These results support data from Bontempo et al. [24] and Corino et al. [13]. Corino et al. [18] also found that weaned piglets fed increasing levels of CLA showed elevated IgG concentrations, suggesting a positive correlation between CLA intake and serum IgG. Moreover, Peng et al. [17] reported that consumption of 0.5 and 1% CLA by lactating sows increased CLA contents in milk and plasma as well as backfat and muscle in their suckling piglets, reflecting maternal CLA transport. However, this study did not analyze the fatty acid profiles of serum and milk. Thus, in the present study, the elevation of IgG concentrations in piglets from sows fed CLA could be attributed to maternal IgG transfer directly to piglets through milk CLA.

Whether or not dietary CLA affects milk yield in lactating sows remains unknown, as milk yield fluctuates depending on breed, parity, suckling intensity, lactational day, and health conditions. Indeed, as this study indirectly measured milk yield using the weigh-suckle-weigh technique [15] based on daily changes in litter BW of sows, there could be differences between real and estimated figures for milk yield. Although there was no difference in daily lactational feed intake between groups, milk yield increased by almost 10% in sows fed CLA compared with sows fed soybean oil. In past studies using lactating cows [50] and lactating ewes [51, 52], rumen-protected or unprotected CLA supplementation increased milk yield. These results are similar to those of the present study, even though it was compared to the milk yield of ruminants other than monogastrics.

On the other hand, CLA reduced milk fat content by about 30%, whereas milk protein, lactose, and solid-not-fat contents were unaffected (Table 5). This result is similar to the data of Harrell et al. [16], who reported that sows fed 1% CLA diet showed 36% reduced milk fat content, whereas milk protein or ash content was unaffected. Moreover, Cordero et al. [14] observed a 14% reduction in crude fat content in milk from sows fed 1% CLA diet compared to those fed control diet. Poulos et al. [53] also reported that 0.5% CLA supplementation from day 40 of gestation until weaning reduced milk fat by 17%. Griinari et al. [54] demonstrated that milk fat depression can occur by either a shortage of precursors for milk fat synthesis or by direct inhibition of milk fat synthesis. Despite the fact that the milk fat content was reduced by CLA supplementation, milk fat yield did not show any significant difference between the treatments. This might be attributable to the offset of milk fat reduction by increased milk yield.

Until now, there has been no report on lactose in lactating sows. In the present study, increased lactose yield due to dietary CLA appeared to be associated with higher circulation of blood glucose to mammary glands for lactose synthesis. Further, although the blood glucose concentration was not significantly different between the treatments (Table 4), a slight increase in blood glucose in sows fed CLA resulted in elevated synthesis of lactose.

In the present study, lower milk fat content due to dietary CLA increased the protein-to-fat ratio. This higher protein-to-fat ratio in sows fed CLA can increase weight at weaning in suckling piglets. Nam and Aherne [55] found that increasing ratios of lysine to DE in weanling piglets linearly increased average daily gain and feed efficiency.

## Conclusion

In conclusion, CLA supplementation to sows during lactation resulted in little change in BW despite increased milk yield, suggesting less mobilization of body stores. Piglets from sows fed CLA showed higher weights at weaning as well as weight gain, but there was no difference in piglet growth after weaning regardless of maternal CLA background. Sows fed CLA also showed reduced serum NEFA and urea nitrogen concentrations as a result of lesser body expenditure, whereas levels of other blood metabolites and thyroid hormone were unaltered. CLA supplementation to sows during lactation increased IgG concentrations not only in sera of sows and piglets but also in milk, implying that CLA may improve the health status and growth of piglets. However, milk produced by sows fed CLA contained lower milk fat content, resulting in a higher protein-to-fat ratio. Further, yield of solid-not-fat in response to dietary CLA consumption remarkably increased due to higher lactose yield.

### Implications

These results show that CLA supplementation to sows increased milk yield without losing BW during lactation, which might positively affect subsequent reproduction and piglet growth. Moreover, soybean oil supplementation to lactating sows resulted in severe BW loss, which might be helpful in reducing BW of obese sows.

## References

[1] Boyd RD, Kensinger RS, Verstegen MWA, Moughun PJ, Schrama JW (1998). Page 71 in The Lactating Sow. Metabolic precursors for milk synthesis.

[2] Mahan DC, Lepine AJ (1991). Effect of weaning weight and associated nursery feeding programs on subsequent performance to 105 kilograms of body weight. J Anim Sci.

[3] Azain MJ (1997). Pages 1–14 in Proc. 13th Annu. Nutrition of the young pig, use of liquid diets.

[4] King RH, Williams IH (1984). The effect of nutrition on the reproductive performance of first-litter sows. 2. Protein and energy intakes during lactation. Anim Prod.

[5] King RH, Dunkin AC (1986). The effect of nutrition on the reproductive performance of first-litter sows. 4. The relative effects of energy and protein intakes during lactation on the performance of sows and their piglets. Anim Prod.

[6] Noblet J, Etienne M (1989). Estimation of sow milk nutrient output. J Anim Sci.

[7] Quesnel H, Etienne M, Père M-C (2007). Influence of litter size on metabolic status and reproductive axis in primiparous sows. J Anim Sci.

[8] Clowes EJ, Aherne FX, Foxcroft GR, Baracos VE (2003). Selective protein loss in lactating sows is associated with reduced litter growth and ovarian function. J Anim Sci.

[9] Thaker MYC, Bilkei G (2005). Lactation weight loss influences subsequent reproductive performance of sows. Anim Reprod Sci.

[10] Kepler CR, Hirons KP, McNeill JJ, Tove SB (1966). Intermediates and products of the biohydrogenation of linoleic acid by *Butyrivibrio fibrisolvens*. J Biol Chem.

[11] Belury MA (2002). Dietary conjugated linoleic acid in health: Physiological effects and mechanisms of action. Annu Rev Nutr.

[12] Pariza MW (2004). Perspective on the safety and effectiveness of conjugated linoleic acid. Am J Clin Nutr.

[13] Corino C, Pastorelli G, Rosi F, Bontempo V, Rossi R (2009). Effect of dietary conjugated linoleic acid supplementation in sows on performance and immunoglobulin concentration in piglets. J Anim Sci.

[14] Cordero G, Isabel B, Morales J, Menoyo D, Pineiro C, Daza A, Lopez-Bote CJ (2011). Conjugated linoleic acid (CLA) during last week of gestation and lactation alters colostrums and milk fat composition and performance of reproductive sows. Anim Feed Sci Technol.

[15] Speer VC, Cox DF (1984). Estimating milk yield of sows. J Anim Sci.

[16] Harrell RJ, Phillips O, Boyd RD, Dwyer DA, Bauman DE (2002). Annu Swine Report 2002. Effects of conjugated linoleic acid on milk composition and baby pig growth in lactating sows.

[17] Peng Y, Ren F, Yin JD, Fang Q, Li FN, Li DF (2010). Transfer of conjugated linoleic acid from sows to their offspring and its impact on the fatty acid profiles of plasma, muscle, and subcutaneous fat in piglets. J Anim Sci.

[18] Corino C, Bontempo V, Sciannimanico D (2002). Effects of dietary conjugated linoleic acid on some aspecific immune parameters and acute phase protein in weaned piglets. Can J Anim Sci.

[19] NRC (1998). Nutrient Requirements of Swine.

[20] AOAC (2005). Official Methods of Analysis (18th Ed).

[21] Sukhija PS, Palmquist DL (1988). Rapid method for determination of total fatty acid content and composition of feedstuffs and feces. J Agric Food Chem.

[22] Mancini G, Carbonara AO, Heremans JF (1965). Immunological quantitation of antigens by single radial immunodiffusion. Immunochemistry.

[23] SAS Institute (2004). Statistics.

[24] Bontempo V, Sciannimanico D, Pastorelli G, Rossi R, Rosi F, Corino C (2004). Dietary conjugated linoleic acid positively affects immunologic variables in lactating sows and piglets. J Nutr.

[25] Sanz M, Lopez-Bote CJ, Menoyo D, Bautista JM (2000). Abdominal fat deposition and fatty acid synthesis are lower and beta-oxidation is higher in broiler chickens fed diets containing unsaturated rather than saturated fat. J Nutr.

[26] Shimomura Y, Tamura T, Suzuki M (1990). Less body-fat accumulation in rats fed a safflower oil diet than in rats fed a beef tallow diet. J Nutr.

[27] Vicente JG, Isabel B, Cordero G, Lopez-Bote CJ (2013). Fatty acid profile of the sow diet alters fat metabolism and fatty acid composition in weanling pigs. Anim Feed Sci Technol.

[28] Park JC, Kim YH, Jung HJ, Moon HK, Kwon OS, Lee BD (2005). Effects of dietary supplementation of conjugated linoleic acid (CLA) on piglets’ growth and reproductive performance in sows. Asian Aust J Anim Sci.

[29] Dugan MER, Alhus JL, Schaefer AL, Kramer JKG (1997). The effect of linoleic conjugated acid on fat to lean repartitioning and feed conversion in pigs. Can J Anim Sci.

[30] Ostrowska E, Muralitharan M, Cross RF, Bauman DE, Dunshea FR (1999). Dietary conjugated linoleic acids increase lean tissue and decrease fat deposition in growing pigs. J Nutr.

[31] Thiel-Cooper RL, Parrish FC, Sparks JC, Wiegand BR, Ewan RC (2001). Conjugated linoleic acid changes swine performance and carcass composition. J Anim Sci.

[32] Wiegand BR, Sparks JC, Parrish FC, Zimmerman DR (2002). Duration of feeding conjugated linoleic acid influences growth performance, carcass traits, and meat quality of finishing barrows. J Anim Sci.

[33] Barnes KM, Winslow NR, Shelton AG, Hlusko KC, Azain MJ (2012). Effect of dietary conjugated linoleic acid on marbling and intramuscular adipocytes in pork. J Anim Sci.

[34] Reese DE, Peo ER, Lewis AJ (1984). Relationship of lactation energy intake and occurrence of postweaning estrus to body and backfat composition in sows. J Anim Sci.

[35] Kirkden RD, Broom DM, Andersen IL (2013). Piglet mortality: Management solutions. J Anim Sci.

[36] McNamara JP, Pettigrew JE (2002). Protein and fat utilization in lactating sows: I. Effects on milk production and body composition. J Anim Sci.

[37] Cabrera RA, Boyd RD, Jungst SB, Wilson ER, Johnston ME, Vignes JL, Odle J (2010). Impact of lactation length and piglet weaning weight on long-term growth and viability of progeny. J Anim Sci.

[38] Bee G (2000). Dietary conjugated linoleic acid consumption during pregnancy and lactation influences growth and tissue composition in weaned pigs. J Nutr.

[39] West DB, Delany JP, Camet PM, Blohm F, Truett AA, Scimeca J (1998). Effects of conjugated linoleic acid on body fat and energy metabolism in the mouse. Am J Physiol.

[40] Ostrowska E, Cross RF, Muralitharan M, Bauman DE, Dunshea FR (2002). Effects of dietary fat and conjugated linoleic acid on plasma metabolite concentrations and metabolic responses to homeostatic signals in pigs. Br J Nutr.

[41] Gutgesell A, Ringseis R, Eder K (2009). Dietary conjugated linoleic acid down-regulates fatty acid transporters in the mammary glands of lactating rats. J Dairy Sci.

[42] Stangl GI (2000). High dietary levels of a conjugated linoleic acid mixture alter hepatic glycerophospholipid class profile and cholesterol-carrying serum lipoproteins of rats. J Nutr Biochem.

[43] Stangl GI (2000). Conjugated linoleic acids exhibit a strong fat-to-lean partitioning effect, reduce serum VLDL lipids and redistribute tissue lipids in food-restricted rats. J Nutr.

[44] Ramsay TG, Evock-Clover CM, Steele NC, Azain MJ (2001). Dietary conjugated linoleic acid alters fatty acid composition of pig skeletal muscle and fat. J Anim Sci.

[45] Mele MC, Cannelli G, Carta G, Cordeddu L, Melis MP, Murru E, Stanton C, Banni S (2013). Metabolism of c9, t11-conjugated linoleic acid (CLA) in humans. PLEFA.

[46] Stangl GI, Müller H, Kirchgessner M (1999). Conjugated linoleic acid effects on circulating hormones, metabolites and lipoproteins, and its proportion in fasting serum and erythrocyte membranes of swine. Eur J Nutr.

[47] Nunes EA, Bonatto SJ, de Oliveira HHP, Rivera NLM, Maiorka A, Krabbe EL, Tanhoffer RA, Fernandes LC (2008). The effect of dietary supplementation with 9-*cis*:12-*trans* and 10-*trans*:12-*cis* conjugated linoleic acid (CLA) for nine months on serum cholesterol, lymphocyte proliferation and polymorphonuclear cells function in Beagle dogs. Res Vet Sci.

[48] Tucker HA (1981). Physiological control of mammary growth, lactogenesis, and lactation. J Dairy Sci.

[49] Beckett GJ, Nicol F, Rae PW, Beach S, Guo Y, Arthur JR (1993). Effects of combined iodine and selenium deficiency on thyroid hormone metabolism in rats. Am J Clin Nutr Suppl.

[50] Bernal-Santos G, Perfield JW, Barbano DM, Bauman DE, Overton TE (2003). Production responses of dairy cows to dietary supplementation with conjugated linoleic acid (CLA) during the transition period and early lactation. J Dairy Sci.

[51] Lock AL, Teles BM, Perfield JW, Bauman DE, Sinclair LA (2006). A conjugated linoleic acid supplement containing *trans*-10, *cis*-12 reduces milk fat synthesis in lactating sheep. J Dairy Sci.

[52] Husvéth F, Galamb E, Gaál T, Dublecz K, Wágner L, Pál L (2010). Milk production, milk composition, liver lipid contents and C18 fatty acid composition of milk and liver lipids in Awassi ewes fed a diet supplemented with protected *cis*-9, *trans*-11 and *trans*-10, *cis*-12 conjugated linoleic acid (CLA) isomers. Small Rum Res.

[53] Poulos SP, Azain MJ, Hausman GJ (2004). Conjugated linoleic acid (CLA) during gestation and lactation does not alter sow performance or body weight gain and adiposity in progeny. Anim Res.

[54] Griinari JM, Chouinard PY, Bauman DE (1997). Trans fatty acid hypothesis of milk fat depression revised.

[55] Nam DS, Aherne FX (1994). The effects of lysine: energy ratio on the performance of weanling pigs. J Anim Sci.

